# Solvent-Evaporation-Induced Synthesis of Graphene Oxide/Peptide Nanofiber (GO/PNF) Hybrid Membranes Doped with Silver Nanoparticles for Antibacterial Application

**DOI:** 10.3390/polym15051321

**Published:** 2023-03-06

**Authors:** Peng He, Minghao Yang, Yu Lei, Lei Guo, Yan Wang, Gang Wei

**Affiliations:** 1 College of Chemistry and Chemical Engineering, Qingdao University, Qingdao 266071, China; 2 Institute of Biomedical Engineering, College of Life Science, Qingdao University, Qingdao 266071, China

**Keywords:** solvent evaporation, graphene membrane, silver nanoparticles, peptide nanofibers, antibacterial application

## Abstract

Designing functional membranes through the collaboration of multi-dimensional nanomaterials is of particular interest in environmental and biomedical applications. Herein, we propose a facile and green synthetic strategy by collaborating with graphene oxide (GO), peptides, and silver nanoparticles (AgNPs) to synthesize functional hybrid membranes with favourable antibacterial effects. GO nanosheets are functionalized with self-assembled peptide nanofibers (PNFs) to form GO/PNFs nanohybrids, in which the PNFs not only improve the biocompatibility and dispersity of GO, but also provide more active sites for growing and anchoring AgNPs. As a result, multifunctional GO/PNFs/AgNP hybrid membranes with adjustable thickness and AgNP density are prepared via the solvent evaporation technique. The structural morphology of the as-prepared membranes is characterized using scanning electron microscopy, transmission electron microscopy, and X-ray photoelectron spectroscopy, and their properties are analyzed by spectral methods. The hybrid membranes are then subjected to antibacterial experiments and their excellent antibacterial performances are demonstrated.

## 1. Introduction

Nanomaterial-based functional membranes have attracted great attention owing to their wide applications in filtration, dialysis, surface modification, tissue engineering, antibacterial materials, and many other fields [1,2,3,4,5]. Typically, various membranes can be prepared through the methods of casting, coating, electrospinning, layer-by-layer self-assembly, evaporation, and vacuum filtration [6,7,8], by which the thickness, pore size, and surface functionality of the fabricated membranes can be tailored effectively. According to different applications, the precursor materials for the fabrication of membranes can range from polymers [9] to biomass nanofibers [10], inorganic nanowires [11], carbon nanofibers [12], carbon nanotubes [13,14], two-dimensional (2D) materials [15,16,17,18], and others.

As a single-layer, biocompatible 2D nanomaterial, graphene, especially in the form of graphene oxide (GO) and reduced graphene oxide (RGO) nanosheets, exhibits high potential for the preparation of functional membranes with excellent electrical, thermal, and mechanical properties [15,19,20]. GO also has good antibacterial activity and the sharp edges of the nanosheets are in direct contact with bacterial cell membranes, thus effectively disrupting the cell membranes and exerting antibacterial effects [21]. Besides these unique structures and properties, GO nanosheets have extra advantages for the preparation of functional membranes. For instance, there are abundant functional groups like -COOH, -OH, and C=O on the surface and edges of GO nanosheets, which provide the possibility for the modification of GO with other nanomaterials to enhance the properties of created GO hybrid membranes [22,23]. In addition, the 2D nanosheet structure makes GO a good support material for conjugation with various nanoparticles, polymers, biomolecules, and biomass nanofibers with high density [24,25,26]. Previously, studies have indicated that GO-based membranes can be utilized for water purification, gas filtration, electronic devices, flexible sensors, battery separators, and antibacterial materials [7,27,28]. Li et al. developed the method of directly mixing acetone and GO aqueous dispersion to prepare GO films with adjustable thickness [29]. Jang et al. studied the adhesion of GO films to several different bacteria, and found that the ability of GO to obtain electrons from bacteria led to the adhesion effect of GO to bacteria. In addition, the adhesion effect of different bacteria is different, and the quality of the GO film also influences the adhesion effect of bacteria [30].

AgNP is a kind of metal nanomaterial that is widely used in medicine, catalysis, food, environment, biosensors, and many others. AgNP, as an excellent broad-spectrum antibacterial material, has a strong antibacterial effect on a variety of bacteria, pathogens, fungi, and so on. The damage mechanism of AgNP to bacteria is that it binds with the bacterial cell membrane to cause membrane damage, interacts with DNA and proteins in bacteria to cause molecular damage, and produces reactive oxygen species, among others. It has also been reported that AgNP can release Ag^+^ to bind with proteins in bacteria to change its active site and three-dimensional structure to provide an additional bactericidal effect [31]. The size of AgNPs is also a key factor affecting their antibacterial properties. Morones et al. explored the antibacterial effect of AgNPs in the range of 1–100 nm [32]. Experiments show that small-sized AgNPs (1–10 nm) can then bind to the cell membrane of bacteria and enter the bacterial interior to play an antibacterial role. This is because the small-sized AgNPs have a larger specific surface area and are more likely to bind to bacteria and enter the bacterial interior. In addition, the surface charge of AgNP also has an impact on its antibacterial activity. Badawyetal et al. evaluated the antibacterial performance of AgNPs with different surface charge intensities, such as the strong electrostatic attraction between negatively charged Bacillus (−37 mV) and positively charged AgNPs. This results in a stronger interaction between AgNPs and bacteria, which improves antibacterial performance. On the other hand, Bacillus will form a certain degree of electrostatic shielding effect on negatively charged AgNPs, which limits the interaction between the two, and thus reduces the antibacterial performance of AgNPs. This study provides ideas for the rational design of AgNPs as antibacterial agents [33].

When applying GO membrane materials for the applications of wound repair, wearable biosensors, water disinfection, and food packaging, one of the key issues that should be considered is the antibacterial performance of the GO membrane used [34,35]. Previous studies have proved that GO nanosheets contributed to the antibacterial effects of related materials through disrupting the cell membrane [36]. However, the antibacterial activity of graphene materials is limited compared with traditional nanomaterials such as silver, copper, and titanium oxide nanoparticles [24,37,38]. In addition, the aggregation and oxide status of GO nanosheets have potential effects on the antibacterial efficiency of GO materials [39]. Therefore, the fabrication of hybrid membranes of GO and highly effective antibacterial nanoparticles is popular for utilizing the combined properties and functions of 2D GO and other materials via synergistic effects. For example, by modifying silver nanoparticles (AgNPs) and copper oxide nanoparticles (CuONPs) onto the surface of GO nanosheets, Menazea et al. chemically prepared functional GO/AgNPs and GO/CuONPs hybrids, respectively. Both GO-based nanohybrids were biocompatible and revealed good inhibitory effects on both *S. aureus* and Gram-negative bacteria [24]. In recent work, Wang et al. designed a simple method to modify GO with CuS nanoparticles to form CuS@GO nanocomposites, and then CuS@GO electrostatically interacted with chitosan (CS) hydrogel to produce CuS@GO-CS antibacterial hydrogel. The antibacterial hydrogel has a large antibacterial effect on Staphylococcus aureus (*S. aureus*) and Escherichia coli (*E. coli*) [40]. In another work, Liang et al. used electrostatic interactions to load zinc oxide quantum dots (ZnO QDs) onto GO nanosheets to synthesize ZnO QDs@GO hybrids, which were further introduced into the as-prepared hydrogel to prepare a hybrid hydrogel with antibacterial activity and wound-healing ability [41].

In this work, we propose a facile solvent evaporation method for the fabrication of GO-based functional hybrid membranes. GO nanosheets are firstly functionalized with self-assembled peptide nanofibers (PNFs), which show high affinity for binding GO to form GO/PNF nanohybrids. The introduction of PNFs onto GO nanosheets not only improves the biocompatibility and dispersity of GO, but also provides more active sites for binding AgNPs via the biomimetic synthesis. By the solvent evaporation, GO/PNF/AgNP hybrid membranes with adjustable thickness and AgNP density are prepared. The created hybrid membranes that exhibit excellent antibacterial activity towards *E. coli* and *S. aureus* owing to the synergistic antibacterial effects of both GO and AgNPs. This facile, green, economic strategy for the fabrication of large-scale GO-based membranes will inspire the design and synthesis of 2D material-based functional membranes for advanced applications.

## 2. Materials and Methods

### 2.1. Materials

All chemicals used in this work are of analytical level. Peptide with the sequence of KIIIIKYWYAF was bought from the SynPeptide Biotechnology Co., Ltd. (Nanjing, China). Monolayered GO aqueous dispersions (GO-1, 10 mg/g) were provided by the Hangzhou Gaoxi Technology Co., Ltd. (Hangzhou, China). D-anhydrous glucose (AR), phosphoric acid, ethanol, glycerol, glutaraldehyde, NaCl, tryptone, and agar powder were purchased from the Shanghai Maclean Biochemical Technology Co., Ltd. (Shanghai, China).

### 2.2. Preparation of PNFs via Self-Assembly

The formation of PNFs is achieved through the peptide self-assembly method. In brief, 1 mg of KIIIIKYWYAF peptide monomer powder was dissolved in 10 mL of water and well dispersed by sonication to configure a 0.1 mg/mL peptide solution. Ultrasound can ensure the full dispersion of peptides. The peptide solution was then incubated in a 37 °C water bath and sampled every 24 h for 3 days to observe the self-assembly status using atomic force microscopy.

### 2.3. Fabrication of GO/PNF/AgNP Hybrid Membrane by Solvent Evaporation

**Scheme 1:**
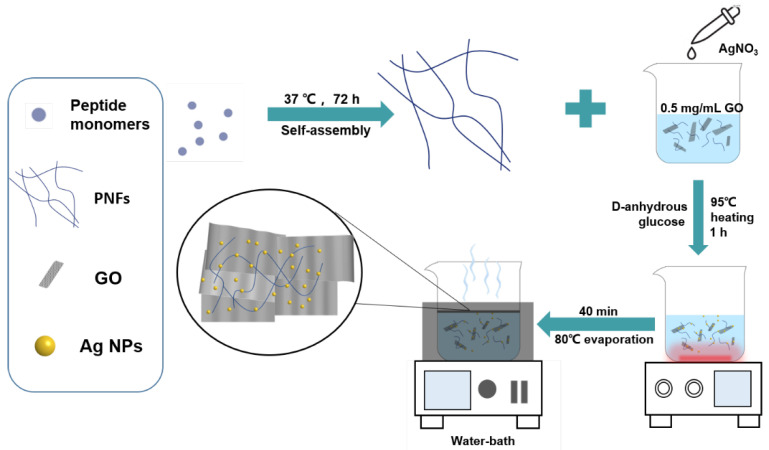
Schematic presentation of the synthesis of GO/PNF/AgNP hybrid membranes.

The preparation of GO/PNF/AgNP hybrid membranes at the air–liquid interface was carried out via the solvent evaporation method, as shown in Scheme 1. Specifically, 2.5 g GO dispersion was diluted to 50 mL to obtain 0.5 mg/mL of GO dispersion. Then, 1 mL of peptide solution and 25 μL of AgNO3 solution were mixed into the GO dispersion. Then, the peptide solution incubated with 1 mL for 3 days and 25 μL AgNO_3_ (0.5 mol/L) solution was mixed into GO dispersion. After the reaction, the mixed solution was placed in a water bath at 80 °C and evaporated for 40 min to obtain GO/PNF/AgNP heterogeneous membranes. The GO/PNF/AgNP hybrid membrane is formed at the water–air interface, the thickness of the hybrid film can be adjusted by changing the evaporation time, and the shape of the hybrid film can be adjusted by changing the loading substrate of the hybrid film.

### 2.4. Characterization Techniques

All AFM samples were characterized by dropping 10 μL of sample onto a newly dissociated mica substrate and drying in air. AFM measurements were performed in air in tap mode using an FM-Nanoview 6800 AFM (FSM-Precision, Feisman Precision Instruments, Suzhou, China). A Tap300Al-G silicon probe (300 kHz, 40 Nm^−1^) was used for AFM image capture. Tap mode images were recorded and analyzed using Gwyddion software (version 2.57). Transmission electron microscopy (Tecnai G2 F20, FEI Co. Hillsborough, OR, USA) was used to observe the structure and morphology of GO/PNF/AgNP suspensions. Scanning electron microscopy (SEM, Regulus 8100, Hitachi Co.Tokyo, Japan) was used to observe the microstructure of GO/PNF/AgNP hybrid membranes. XPS characterization of the samples was performed on a PHI 5000 VersaProbe III spectrometer (PHI-5000 Versaprobe III, UlVAC-PHI Co., Kanagawa, Japan).

### 2.5. Antibacterial Tests

Before the antimicrobial test, GO/PNF/AgNP hybrid membranes attached to PET were obtained by fishing up the GO/PNF/AgNP hybrid membranes in the beaker using a 1 cm diameter PET disc.

The bactericidal activities of all of the samples were investigated against *E. coli* (ATCC25922) and *S. aureus* (ATCC25923) bacteria as Gram-negative and Gram-positive models, respectively. Then, 1.5 g of tryptone, 0.75 g of yeast extract, and 1.5 g of NaCl were dissolved in 150 mL of deionized water to form a bacterial culture solution, which was sterilized using an autoclave (LDZX-50KBS, Shanghai Shenan Medical Equipment Factory Shanghai, China) for 30 min and then added to bacteria and placed in a 37 °C constant temperature shaking incubator (THZ-98A, Shanghai Yiheng Scientific Instruments Co. (THZ-98A, Shanghai Yiheng Scientific Instruments Co., Ltd., Shanghai, China) for 14 h. In the adhesion experiment, the concentration of the above bacterial culture solution was measured using a bacterial turbidity meter (WGZ-XY, Hangzhou Qiwei Instruments Co., Ltd., Hangzhou, China) and diluted to 1 × 10^6^ CFU mL^−1^. Then, 50 mL of 1 × 10^6^ CFU mL^−1^ *E. coli* solution was transferred to a 200 mL conical flask. The blank wafer and the wafer covered with the hybrid membrane (1 cm in diameter) were sterilized using UV light for 30 min and, after sterilization, they were placed in the above 200 mL conical flask and incubated in a constant temperature shaking incubator at 37 °C for 24 h(In the experiment, 4 bottles of bacterial culture solution were configured, the No. 1 bottle was added with blank silicon wafers, and the No. 2–4 bottles were added with GO/PNF/AgNP hybrid film). After incubation, the samples were removed from the bacterial solution, washed with distilled water to remove excess bacterial solution, and then immersed in fixative (2.5% glutaraldehyde, solvent: 0.1 M PBS solution, pH = 7.4) for 6 h. Finally, the samples were dehydrated through a series of ethanol solutions of different concentrations (30, 40, 50, 60, 70, 80, 90, 95, and 100%, respectively) for 3 min, air-dried overnight, and then observed using SEM.

For the antimicrobial loop experiment, 100 μL of *E. coli* (1 × 10^6^ CFU mL^−1^) solution was uniformly coated in the solid medium. Then, the hybrid membranes with different AgNP loads were placed in the culture medium and co-cultured with bacteria (four kinds of hybrid membranes with different AgNP loads were tested, and the blank PET membrane was used as the control group). Three sets of parallel experiments were performed simultaneously and, after 24 h, a gel imager (JY04S-3E, Beijing Junyi Oriental Electrophoresis Equipment Co., Ltd., Beijing, China) was used to observe the bacterial growth.

## 3. Results and Discussion

### 3.1. Synthesis and Characterization of GO/PNF Nanohybrids

When the peptide AFM samples were prepared at different times, 10 μL of 0.1 mg/mL peptide solution was dropped on the mica sheet, and the mica sheet was completely dried for testing. When the AFM sample of GO was prepared, 10 μL of 0.1 mg/mL of GO dispersion was dropped on the mica sheet, and the mica sheet was completely dried for testing. During the preparation of GO/PNF samples, 1 mL of 0.1 mg/mL polypeptide solution and 10 mL of 0.1 mg/mL GO solution were thoroughly mixed and stirred for 2 h, then 10 μL drops were added to mica sheet, and the mica sheet was completely dried for testing.

**Figure 1:**
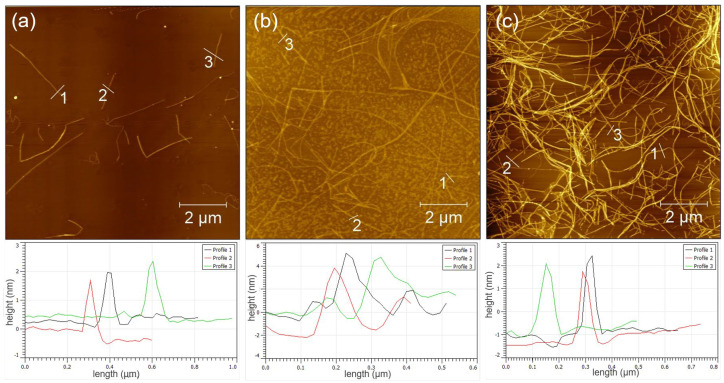
AFM images and cross−sectional analysis of KIIIIKYWYAF peptide nanofibers at different incubation times: (**a**) 24 h, (**b**) 48 h, and (**c**) 72 h.

**Figure 2:**
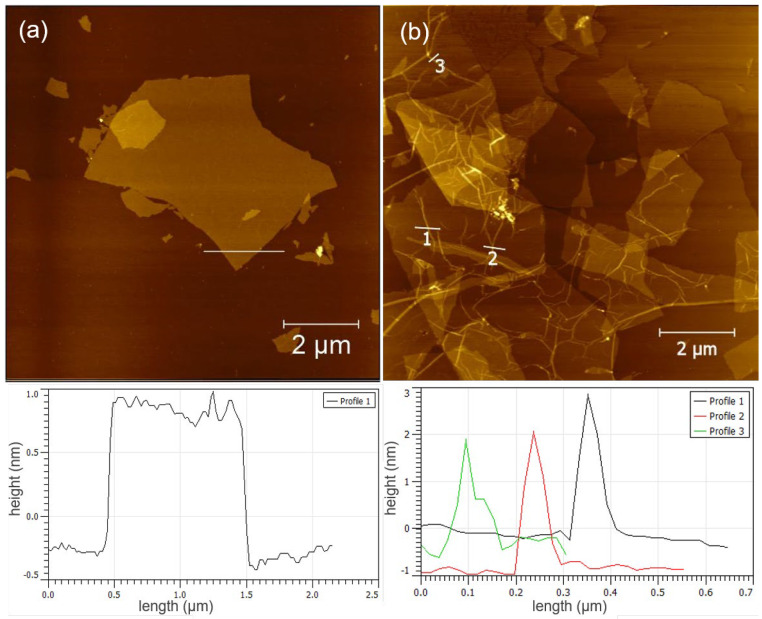
Morphological and structural features of GO and GO/PNFs: (**a**) AFM images and cross-sectional analysis of GO; (**b**) AFM images and cross−sectional analysis of GO/PNFs.

KIIIIKYWYAF sequence peptide design can form with PNFs; KIIIIKYWYAF consists of two typical peptide motifs KIIIIK and YWYAF. The KIIIIK motif is known to be a typical β-folded amino acid sequence, while YWYAF contains a large number of aromatic groups, so it can interact with the graphene surface through π–π interaction, resulting in a strong interaction without the involvement of other substances [42]. Figure 1 shows the self-assembly of polypeptide molecules at different time points. At 24, 48, and 72 h incubation time, 10 μL of the peptide solution was diluted 10 times and then 10 μL of the sample was dropped on the mica sheet for AFM observation. It was found that a small number of peptide nanofibers appeared at 24 h incubation time and the fiber length was about 3–4 μm (Figure 1a). When the peptide was incubated for 48 h, there were some polypeptide aggregates in the solution, but some typical peptide nanofiber structures had begun to form. AFM images showed the coexistence of peptide aggregates and PNF as a whole. The number and length of peptide nanofibers increased, but there were still a large number of peptide monomers that had not completed self-assembly (Figure 1b). When the peptide was incubated for 72 h, a large number of peptide nanofibers appeared, with a long fiber length and a stable diameter of about 3 nm (Figure 1c). Figure 1 shows the self-assembly of a possible polypeptide monomer to form PNFs, in which it is easy to understand the transformation process of the polypeptide molecule, which forms well-formed polypeptide nanofibers in solution and exhibits long-term stability. Therefore, we believe that the formation of PNFs is dynamic and the self-assembly process is related to the incubation time of peptide molecules. However, a number of other factors such as temperature, ionic strength, and peptide concentration are also key to the nano structural morphology of the peptide created. PNFs remained in good shape after 30 days of continuous storage at room temperature. Figure 2a shows the AFM image of the GO nanosheet. It can be seen that the size of the GO nanosheet is about 4 μm and the surface is flat. Through atomic force microscope cross-section analysis, the height of the GO plate was measured to be about 1 nm. Figure 2b shows the GO/PNF nanosheet formed by an even mixture of GO and PNFs. The number 1–3 in the figure shows the peptide nanofibers bound on GO nanosheets, and the results of interface analysis are consistent with those in the above.

PNFs formed by the KIIIIKYWYAF peptide have a large number of aromatic groups, which can interact with GO nanosheets to produce a strong specific binding force, thus promoting the binding of a large number of PNFs with GO nanosheets.

The binding of PNFs facilitates the attachment of GO nanosheets to each other; meanwhile, the PNFs contain a large number of active centers that can adsorb metal ions through classical coordination interactions.

### 3.2. Synthesis and Characterization of GO/PNF/AgNP Hybrid Membranes

**Figure 3:**
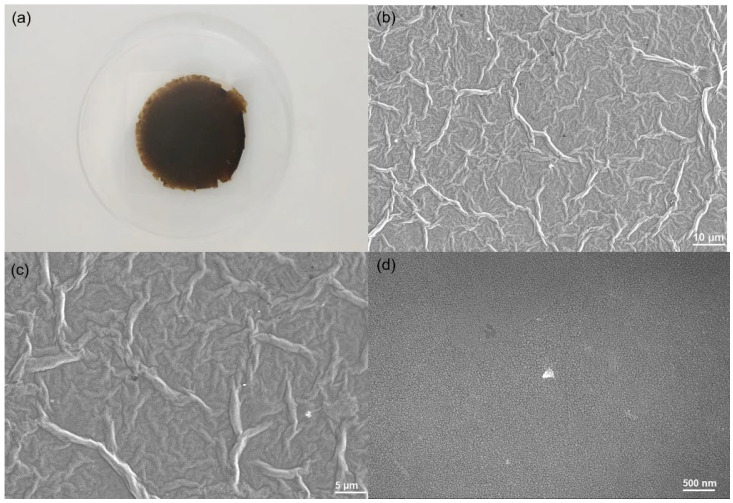
Macroscopic images and SEM images of GO/PNF/AgNP hybrid membranes: (**a**) macroscopic images of GO/PNF/AgNP hybrid membranes. (**b**–**d**) SEM images of GO/PNF/AgNP hybrid membranes at different magnifications.

As shown in Figure 3a, evaporation forms a GO/PNF/AgNP hybrid film loaded on the PET film. Owing to the use of a beaker evaporation, the resulting hybrid film is round. The GO/PNF/AgNP hybrid film is dark brown with a flat surface and diameter of about 6 cm. A large number of GO/PNF/AgNP nanomaterials were self-assembled at the air–liquid interface during solution evaporation, resulting in the formation of GO/PNF/AgNP hybrid membranes. Figure 3b and c show that the GO/PNF/AgNP hybrid membrane has a unique folded morphology. In the magnified SEM image, it can be seen that the AgNPs are densely distributed over the whole graphene surface (Figure 3d).

**Figure 4:**
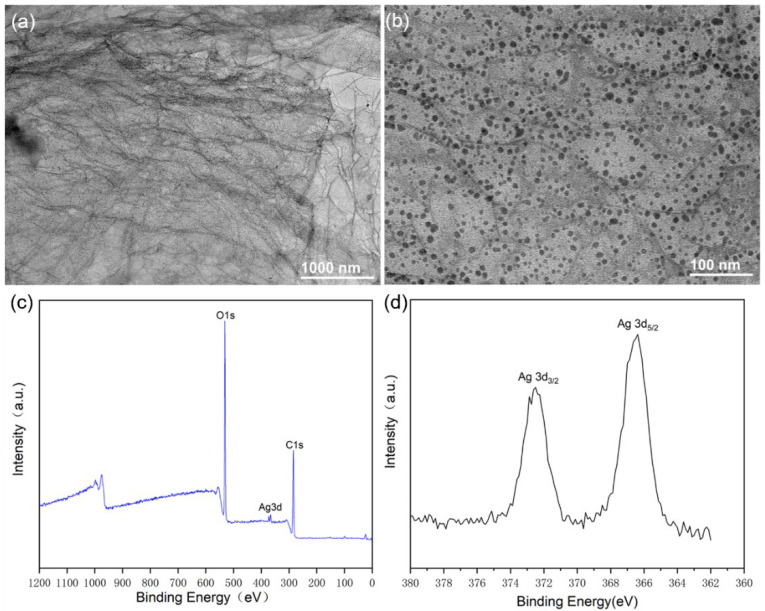
(**a**,**b**) TEM images of GO/PNF/AgNP suspensions. (**c**,**d**) XPS spectra of GO/PNF/AgNP hybrid membranes.

To characterize the distribution of NPs and PNFs before the self-assembly of the GO/PNF/AgNP hybrid membrane, TEM images were taken of the reaction solution before evaporation into the membrane. From Figure 4a, it can be clearly seen that the lamellar connected GO nanosheets and a large number of black nanoparticles are generated on the surface. In the further magnified TEM image (Figure 4b), it can be found that PNFs and NPs are uniformly loaded on the surface of GO nanosheets, with a larger number of NPs being observed around PNFs, which further proves the interaction between PNFs and NPs. To further prove that the generated NPs are AgNPs, the GO/PNF/AgNP hybrid membranes were lyophilized and analyzed by XPS. The obtained XPS spectrum from Figure 4c indicates that the generated GO/PNF/AgNP heterogeneous membranes include C1s, N1s, O1s, and Ag3d. Moreover, the detailed XPS spectrum of Ag3d (Ag3d_5/2_ at 367 eV and Ag3d_3/2_ at 373 eV) provides direct evidence for the formation and modification of AgNPs on GO nanosheets (Figure 4d). Based on the above results, we suggest that the GO/PNF/AgNP hybrid membranes were created successfully.

**Figure 5:**
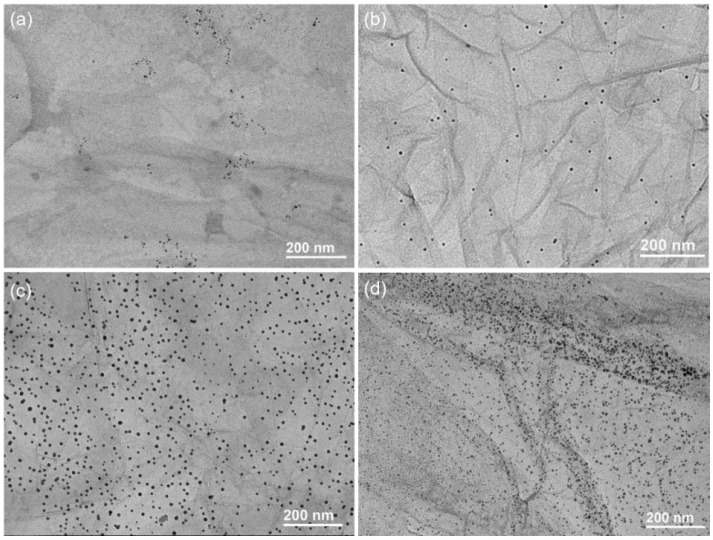
TEM images of GO/PNF/AgNP hybrid membrane suspensions with different AgNO_3_ additions: (**a**) 15, (**b**) 25, (**c**) 50, and (**d**) 75 μL, respectively.

In order to further investigate the relationship between the addition of AgNO_3_ solution and the addition of AgNPs on the surface of GO nanosheets, TEM characterization of GO/PNF/AgNP hybrid membrane suspensions with different AgNO_3_ additions was carried out. From Figure 5a, it can be seen that the addition of 15 μL just forms a small portion of AgNPs on the surface and the distribution was not uniform. In Figure 5b, the distribution of AgNPs becomes uniform, and Figure 5c shows that the AgNPs on the GO nanosheets are uniformly distributed and the density becomes larger; further, in Figure 5d, the density of AgNPs on the GO nanosheets increases further. Therefore, we can regulate the density of AgNPs on GO nanosheets by adjusting the addition amount of AgNO_3_ solution, through which the antibacterial function of the created hybrid membranes can be tailored effectively.

### 3.3. Antibacterial Performacne of the Fabricated GO/PNFs/AgNPs Hybrid Membranes

We further investigated the antibacterial properties of the prepared GO/PNF/AgNP hybrid membranes and characterized their antibacterial properties by adhesion experiments and inhibition circle experiments.

**Figure 6:**
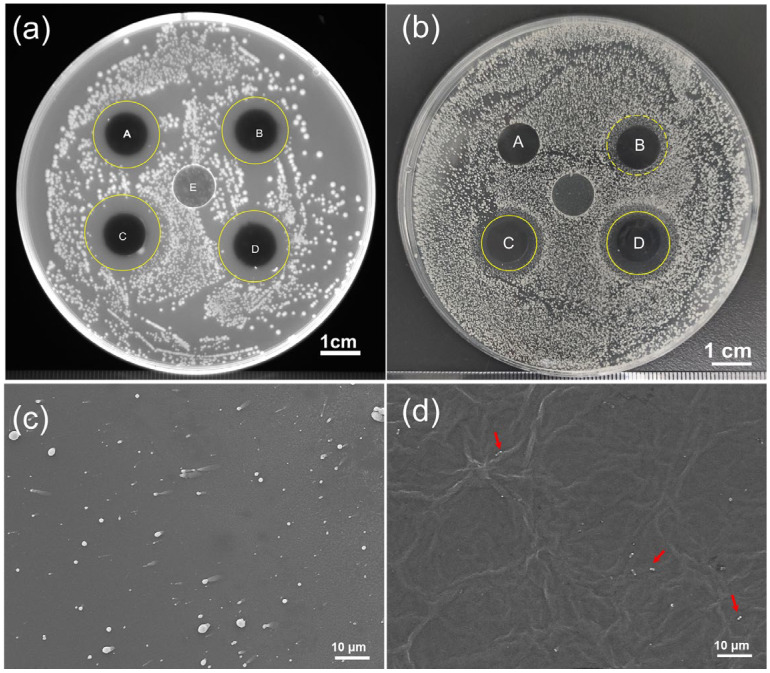
(**a**) The bacteriostatic effect of the GO/PNF/AgNP hybrid membrane on *E. coli*. (**b**) The bacteriostatic effect of the GO/PNF/AgNP hybrid membrane on *S. aureus* (A–D: the content of AgNO_3_ in the hybrid membrane is 15, 25, 50, and 75 μL, respectively; E: blank SEM film without the hybrid membrane) after the attachment experiment. (**c**) Blank silicon wafer and (**d**) silicon wafer covered with the hybrid film.

Figure 6a,b shows the bacteriostatic effect of hybrid membrane with different doses of AgNO_3_ on *E. coli* and *S. aure*us, respectively. Sample E is a blank PET film without bacteriostasis. In Figure 6a, the bacteriostatic effect of sample A–D on *E. coli* increased with the increase in the amount of AgNO_3_, and the diameters of the bacteriostatic ring were 1.63, 1.66, 1.75, and 1.87 cm, respectively. In Figure 6b, A–D also show an obvious inhibitory effect on *S. aure*us with the increase in AgNO_3_. No obvious bacteriostatic circle was observed in sample A, the bacterial group around sample B was sparse, and the bacteriostatic circle was not apparent. Samples C and D showed obvious bacteriostatic circles, with diameters of 1.33 and 1.50 cm, respectively. The results showed that the GO/PNF/AgNP hybrid membrane exhibited an obvious inhibitory effect on both *S. aure*us and *E. coli*, and the inhibitory effect for *E. coli* is stronger under the same conditions. In the adhesion experiment shown in Figure 6c, a denser distribution of *E. coli* can be clearly seen in the SEM image of the silicon wafer surface without the hybridized membrane, while a small number of *E. coli* (partially marked with red arrows) are observed in the SEM image of the hybridized membrane covered in Figure 6d. Through the experimental control, GO/PNF/AgNP hybrid membranes showed obvious performance of inhibiting the growth of bacterial colony. All of the above results showed that the synthesized hybrid membrane had a good antibacterial effect.

GO and AgNPs in GO/PNF/AgNP hybrid membranes have a synergistic antibacterial effect. In previous studies, it was found that the attachment of AgNPs to the microbial membrane resulted in irreversible morphological changes in the structure of the cell membrane, and further interaction with the cell membrane induced local membrane perforation, which led to the internalization of AgNPs and affected cell activity [43,44]. In addition, Ag^+^ can be produced from AgNPs to further improve the antibacterial performance [45]. However, the simple aggregation of AgNPs may lead to the decrease in its antibacterial activity, so the GO membrane, as the loading substrate of AgNPs, can effectively avoid the aggregation of AgNPs and improve the life of antibacterial activity. On the other hand, GO and its derivatives can induce the degradation of bacterial inner and outer cell membranes and reduce the viability of bacteria [21]. Although pure GO in this study may not have significant antibacterial properties, they play a positive role in bacterial adhesion to the surface of the GO/PNF/AgNP hybrid membrane, which can significantly increase the interaction between AgNPs and the bacterial surface. Therefore, PNFs contribute to the increase in biocompatibility and enhance the connection between the GO piece and piece from the morphological structure of the stable GO/PNF/AgNP composite membrane. Based on the synergistic effect of AgNPs and GO on antibacterial properties, the prepared GO/PNF/AgNP composite membrane has good antibacterial properties against *E. coli* and *S. aureus*.

## 4. Conclusions

In summary, we designed a simple thermal reduction and solvent evaporation to drive a self-assembly method to prepare GO-based thin films. The size, thickness, and loading of metal nanoparticles of the membranes can be tuned by the amount of reagent addition, reaction time, and size of reaction vessels. The shape of the hybridized membrane can also be regulated by selecting different covering substrates. GO/PNF/AgNP hybrid membranes of different sizes have good hydrophilicity and loading effects and can be stably fixed on the substrate for a long time. Owing to the synergistic antibacterial effects of both GO and AgNPs, the hybrid membranes exhibited desirable antibacterial activity towards *E. coli* and *S. aureus*. Moreover, the membranes also show promising prospects for biomedical applications owing to the good biocompatibility of PNFs. Overall, the proposed green, facile, and cost-effective strategy for the fabrication of GO/PNF/AgNP membranes will inspire the design and synthesis of 2D-material-based functional membranes toward promoting antibacterial performances.

## Data Availability

Not applicable.
